# Coexistence and competition of sulfate-reducing and methanogenic populations in an anaerobic hexadecane-degrading culture

**DOI:** 10.1186/s13068-017-0895-9

**Published:** 2017-09-05

**Authors:** Ting-Ting Ma, Lai-Yan Liu, Jun-Peng Rui, Quan Yuan, Ding-shan Feng, Zheng Zhou, Li-Rong Dai, Wan-Qiu Zeng, Hui Zhang, Lei Cheng

**Affiliations:** 1 Key Laboratory of Development and Application of Rural Renewable Energy, Biogas Institute of Ministry of Agriculture, Section 4-13, Renmin South Road, Chengdu, 610041 People’s Republic of China; 2 Key Laboratory of Environmental and Applied Microbiology, Chengdu Institute of Biology of Chinese Academy of Sciences, Section 4-9, Renmin South Road, Chengdu, 610041 People’s Republic of China; 3 Environmental Microbiology Key Laboratory of Sichuan Province, Section 4-9, Renmin South Road, Chengdu, 610041 People’s Republic of China; 4 State Key Laboratory of Environmental Geochemistry, Institute of Geochemistry, Chinese Academy of Sciences, 99 Lincheng West Road, Guanshanhu District, Guiyang, 550081 People’s Republic of China; 5 Anhui Normal University, 1 Beijing East Road, Wuhu, 241000 People’s Republic of China

**Keywords:** Oil reservoirs, Hydrocarbon degradation, Methane production, Sulfate reduction, Microbial interactions

## Abstract

**Background:**

Over three-fifths of the world’s known crude oil cannot be recovered using state-of-the-art techniques, but microbial conversion of petroleum hydrocarbons trapped in oil reservoirs to methane is one promising path to increase the recovery of fossil fuels. The process requires cooperation between syntrophic bacteria and methanogenic archaea, which can be affected by sulfate-reducing prokaryotes (SRPs). However, the effects of sulfate on hydrocarbon degradation and methane production remain elusive, and the microbial communities involved are not well understood.

**Results:**

In this study, a methanogenic hexadecane-degrading enrichment culture was treated with six different concentrations of sulfate ranging from 0.5 to 25 mM. Methane production and maximum specific methane production rate gradually decreased to 44 and 56% with sulfate concentrations up to 25 mM, respectively. There was a significant positive linear correlation between the sulfate reduction/methane production ratio and initial sulfate concentration, which remained constant during the methane production phase. The apparent methanogenesis fractionation factor (*α*_app_) gradually increased during the methane production phase in each treatment, the *α*_app_ for the treatments with lower sulfate (0.5–4 mM) eventually plateaued at ~1.047, but that for the treatment with 10–25 mM sulfate only reached ~1.029. The relative abundance levels of *Smithella* and *Methanoculleus* increased almost in parallel with the increasing sulfate concentrations. Furthermore, the predominant sulfate reducer communities shifted from *Desulfobacteraceae* in the low-sulfate cultures to *Desulfomonile* in the high-sulfate cultures.

**Conclusion:**

The distribution of hexadecane carbon between methane-producing and sulfate-reducing populations is dependent on the initial sulfate added, and not affected during the methane production period. There was a relative increase in hydrogenotrophic methanogenesis activity over time for all sulfate treatments, whereas the total activity was inhibited by sulfate addition. Both *Smithella* and *Methanoculleus*, the key alkane degraders and methane producers, can adapt to sulfate stress. Specifically, different SRP populations were stimulated at various sulfate concentrations. These results could help to evaluate interactions between sulfate-reducing and methanogenic populations during anaerobic hydrocarbon degradation in oil reservoirs.

**Electronic supplementary material:**

The online version of this article (doi:10.1186/s13068-017-0895-9) contains supplementary material, which is available to authorized users.

## Background

Crude oil is the most relied-upon source of energy supply worldwide, accounting for 32.9% of global energy consumption [1]. However, the oil recovery efficiency in most oilfields around the world is commonly less than 40% [2], indicating that a larger amount of oil trapped in subsurface reservoirs has not been efficiently explored, even with advanced techniques of enhanced oil recovery (EOR) [3]. Geological and geochemical studies suggest that the vast majority of oil reservoirs have undergone biodegradation over millennia, and oil biodegradation in most reservoirs must indeed have been anaerobic in nature [4–6]. The methanogenic degradation of crude oil to methane appears to commonly proceed in oil reservoirs over geological time-scales, and biogenic methane may account for over 20% of global conventional recoverable gas resources [5, 7, 8]. Many microbial studies in the past twenty decades have demonstrated that aliphatic and aromatic hydrocarbons can be biodegraded in anoxic environments where nitrate, sulfate, and metal ions can function as terminal electron acceptors; when those electron acceptors are limited, the hydrocarbons can be further converted to methane and carbon dioxide in a process known as methanogenic crude oil biodegradation [9]. The methanogenic process has also been observed in oil fields [10–13], which suggests the in situ bioconversion of hydrocarbons in oil reservoirs. As anaerobic microbial communities can play significant roles in increasing the amount of energy recovered and reducing the exploitation cost by converting petroleum hydrocarbons into methane and carbon dioxide [10, 14, 15], an alternative technique called microbial enhanced energy recovery (MEER) was proposed [14, 16]. However, this process takes several months and even years under laboratory incubation conditions, and may also require much longer time to complete in field-scale experiments for environmental constraints [10, 11, 17]. In addition, the microbial communities commonly utilize a small fraction of crude oil [10, 11, 17]. Hence careful exploration of the oil degradation rate and efficiency are extremely important when considering the prospects of the technique in the future [16].

Methanogenic degradation of hydrocarbons to methane requires at least two different groups of microorganisms (syntrophic bacteria and methanogenic archaea) due to thermodynamic constraints [18]. Hydrocarbon is first degraded and converted into hydrogen and/or acetate by syntrophic bacteria; the byproducts are further converted into methane by methanogens [18]. In addition to methanogenic and syntrophic consortia, sulfate-reducing prokaryotes (SRPs), which were first reported in oil reservoirs 90 years ago [19], have also been identified in diverse geographical oil reservoirs and laboratory cultures capable of methanogenic hydrocarbon degradation [12, 20–24]. SRPs not only utilize simple compounds such as H_2_/CO_2_ and acetate but also degrade a variety of complex organic chemicals, such as hydrocarbons and fatty acids, to produce CO_2_ and/or acetate [25, 26]. In the presence of sulfate, SRPs are believed to outcompete methanogens in most cases due to their high affinities for common substrates, which divert carbon and the electron flow of organic compounds away from methanogenesis to sulfidogenesis [27–29]. When sulfate is absent or limiting, some SRPs are also able to survive by cooperating with methanogens and syntrophic bacteria [25]. Sulfate is a common constituent in oil fields, with concentrations ranging from zero to several thousand milligrams per liter [24, 30], and is a key factor affecting the activity of SRPs and the interactions between methanogenic populations and SRPs. Several studies of the methanogenic degradation of alkanes have reported that sulfate concentrations less than 2–5 mM can enhance alkane-dependent methanogenesis, while high concentrations of sulfate (10–22 mM) decreased the methanogenesis rate [11, 15, 31]. However, the interactions between methanogenic populations and SRPs during alkane degradation have not yet been fully exploited, and the contribution of them to alkane degradation has not been quantitatively described.

Previous studies have revealed that a series of novel uncultured bacteria dominated in methanogenic alkane-degrading cultures enriched from oil fields and oil-contaminated sediments [20, 21]. Among them, *Syntrophaceae*-affiliated members have always been detected [13, 15, 32–35] and have been identified as key players in alkane activation and oxidation [33, 34]. Metagenomic and single-cell genomic analysis suggested that *Syntrophaceae* activates the initial degradation of hexadecane by fumarate addition [36–38], which was further demonstrated by direct detection of methyl pentadecyl succinic acid and methyl tetradecyl succinic acid in methanogenic cultures amended with hexadecane and pentadecane, respectively [39]. The archaeal communities in the above-mentioned cultures were composed of aceticlastic and hydrogenotrophic methanogens, but the phylogenetic compositions and abundance levels of two types of methanogens varied in different cultures [11, 13, 15, 32–35, 40]. In addition, SRPs, such as *Desulfovibrionales*, *Desulfuromonadales,* and *Desulfobacterales*, also coexisted in *Syntrophaceae*-dominant, alkane-degrading cultures [15, 32, 34, 35]. However, it remains unclear how methanogenic microbial communities, including syntrophic alkane degraders and methane producers, respond to sulfate stress and which phylogenetic groups of SRPs are responsible for sulfate reduction at different sulfate concentrations.

Therefore, we incubated a highly enriched, methanogenic hexadecane-degrading culture with amendment of different sulfate concentrations (0.5, 2, 4, 10, 15, and 25 mM) to describe the carbon and electron flow between methane production and sulfate reduction during anaerobic hexadecane degradation and to evaluate the effects of sulfate on the methane production pathway. Culture-independent molecular techniques (terminal restriction fragment length polymorphism [T-RFLP], cloning and MiSeq sequencing of 16S rRNA genes) were applied to assess the shifts in microbial community compositions.

## Results

### Methane production and sulfate reduction

**Fig. 1:**
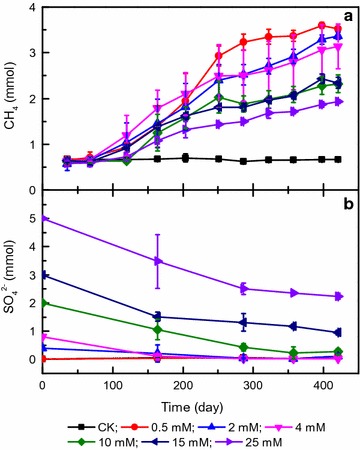
Methane production (**a**) and sulfate consumption (**b**) in cultures amended with different sulfate concentrations. CK, culture without hexadecane and sulfate addition; 0.5, 2, 4, 10, 15, and 25 mM, represent cultures amended with hexadecane and 0.5, 2, 4, 10, 15, and 25 mM sulfate, respectively. Values represent means and standard errors (*n* = 2–3 replicates)

Methane production began after a lag phase of approximately 120 days and increased over a total incubation period of 421 days in the cultures in which hexadecane and variable concentrations of sulfate were present (Fig. 1a). A maximum methane production of 3.52 ± 0.02 mmol was observed in the culture with 0.5 mM sulfate. Methane production gradually decreased with increasing sulfate concentration and dropped to 1.93 ± 0.04 mmol in the 25 mM sulfate culture (Fig. 1a). The maximum specific methane production rate (*μ*_max_) declined from 0.41 ± 0.005 month^−1^ in the 0.5 mM sulfate culture to 0.20 ± 0.02 month^−1^ in the 25 mM sulfate culture (Table 1). In contrast, the hexadecane- and sulfate-free culture only accumulated an average of 0.66 mmol of methane during the entire incubation period (Fig. 1a). The amount of residual hexadecane decreased from 0.074 ± 0.052 mmol for the 0.5 mM sulfate culture to 0.015 ± 0.006 mmol for the 25 mM sulfate culture at the end of the incubation period (Additional file 1: Figure S1). Correspondingly, sulfate consumption increased from 0.08 ± 0.01 mmol for the 0.5 mM sulfate culture to 2.66 ± 0.11 mmol for the 25 mM sulfate culture (Fig. 1b; Table 1). A trace amount of sulfate (an average of 0.1 mM) was detected in the control culture (data not shown). No volatile fatty acid concentrations were observed above the detection limit of this method (approximately 100 μM) (data not shown).

**Table 1:** Growth properties of the M82 culture incubated at different sulfate concentrations

Initial sulfate concentration (mM)	Hexadecane consumption (mmol)	Sulfate consumption (mmol)^a^	Methane production (mmol)^b^	Recovery of hexadecane carbon (%)	Maximum specific methane production rate (month^−1^)	(Sulfate reduction + methane production)/hexadecane consumed
0.5	0.27 ± 0.05	0.08 ± 0.01	2.86 ± 0.02	91.7 ± 17.3	0.41 ± 0.005	11.23 ± 2.12
2	0.29 ± 0.03	0.19 ± 0.08	2.71 ± 0.01	82.1 ± 7.5	0.27 ± 0.08	10.06 ± 0.92
4	0.29 ± 0.06	0.67 ± 0.01	2.48 ± 0.49	89.7 ± 5.1	0.30 ± 0.09	10.99 ± 0.63
10	0.27 ± 0.05	1.62 ± 0.1	1.61 ± 0.24	101.0 ± 9.7	0.23 ± 0.04	12.37 ± 1.18
15	0.31 ± 0.01	1.95 ± 0.08	1.68 ± 0.13	96.9 ± 5.5	0.22 ± 0.02	11.87 ± 0.67
25	0.33 ± 0.01	2.66 ± 0.11	1.27 ± 0.04	98.1 ± 3.6	0.20 ± 0.02	12.01 ± 0.45

^a^The detected sulfate at day 421 minus the background sulfate (0.096 mmol)

^b^The detected methane at day 421 minus the background methane (0.66 mmol)

**Fig. 2:**
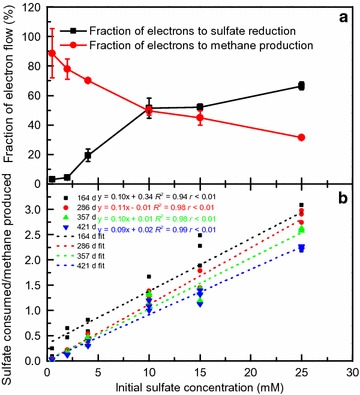
Effects of sulfate on electron flow (**a**) and ratio of sulfate reduction/methane production (**b**). Values represent means and standard errors (*n* = 2–3 replicates)

Assuming total degradation of hexadecane via methanogenesis and/or sulfidogenesis (Eqs. 2 and 3), the percentage of hexadecane carbon recovery averaged 93.9 ± 9.0% in the 82.1–101.0% range (Table 1). The carbon balance of hexadecane consumption linked to sulfidogenic and methanogenic degradation was further evaluated. For every 1 mol of hexadecane degraded, an equivalent 12.25 mol of combined methane production and sulfate consumption occurred (Eqs. 2 and 3), consistent with the observed average values of 11.49 ± 1.11 in the 10.06–12.37 range (Table 1). However, the estimated contribution of hexadecane degradation through methanogenesis decreased from 88.6 ± 16.7% in the 0.5 mM sulfate culture to 31.6 ± 1.1% in the 25 mM sulfate culture (Fig. 2a). The decrease in electron flow to methane production was completely compensated by an increase in electron flow to sulfate reduction, which increased from 3.1 ± 0.6% in the 0.5 mM sulfate culture to 66.4 ± 2.6% in the 25 mM sulfate culture (Fig. 2a). The ratio of these two estimates, i.e., the sulfate reduction to methane production ratio, correlated positively with the initial sulfate concentrations (*R* ^2^ = 0.94–0.99, *p* < 0.01) (Fig. 2b). Further analysis revealed that the differences in the regression results were quite small between 164 and 421 days of incubation; the values of the slope coefficients (0.09–0.11) were also similar (Fig. 2b).

### Isotopic fractionation of methane incubated at different sulfate concentrations

**Fig. 3:**
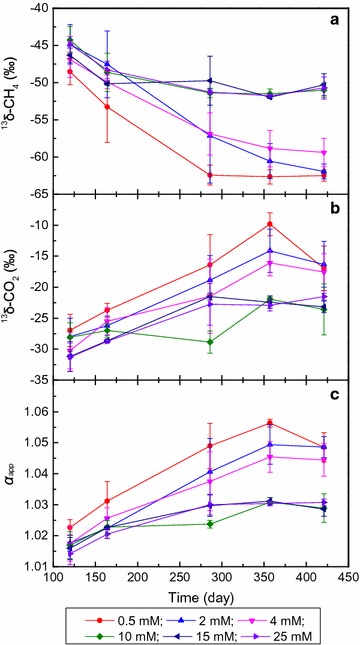
Values of *δ* ^13^CH_4_ (**a**), *δ* ^13^CO_2_ (**b**) and apparent fractionation factors (*α*_C_) (**c**). CK, culture without hexadecane and sulfate addition; 0.5, 2, 4, 10, 15, and 25 mM represent cultures amended with hexadecane and 0.5, 2, 4, 10, 15, and 25 mM sulfate, respectively. Values represent means and standard errors (*n* = 2–3 replicates)

Isotopic analysis revealed that the δ^13^C values of methane (δ^13^CH_4_) generated were similar at the early stage of methane production (120 days), i.e., averaging −45.9 ± 2.2‰ (Fig. 3a). With increasing CH_4_ accumulation, δ^13^CH_4_ continued to decrease and plateaued at −61.5 ± 1.7 and −50.7 ± 1.2‰ for the 0.5–4 mM and 10–25 mM sulfate cultures, respectively, after 421 days of incubation (Fig. 3a). The CO_2_ concentrations measured in the headspace were used for isotopic analysis, since CO_2_ rather than bicarbonate is the active substrate for hydrogenotrophic methanogenesis [41]. The δ^13^C value of carbon dioxide (δ^13^CO_2_) was −28.7 ± 2.8‰ in all sulfate addition cultures after 120 days of incubation. The δ^13^CO_2_ values continued to increase during incubation and plateaued at −17.0 ± 3.4 and −22.8 ± 1.9‰ for the 0.5–4 and 10–25 mM sulfate cultures, respectively (Fig. 3b). According to Eq. 1, reported by Whiticar et al. [42] the δ^13^C values of CH_4_ and CO_2_ were used to compute the apparent fractionation factors (*α*_app_). The *α*_app_ values initially remained at 1.017 ± 0.004 during the early stage and increased to 1.047 ± 0.004 in the 0.5–4 mM sulfate cultures. However, the *α*_app_ value had only increased to 1.029 ± 0.003 in the 10–25 mM sulfate cultures at the end of incubation (Fig. 3c).

1 $$ \alpha_{\text{app}} = (\delta^{ 1 3} {\text{CO}}_{ 2} + 1000)/(\delta^{ 1 3} {\text{CH}}_{ 4} + 1000) $$

### Microbial community structures and functions

#### Archaeal community

**Fig. 4:**
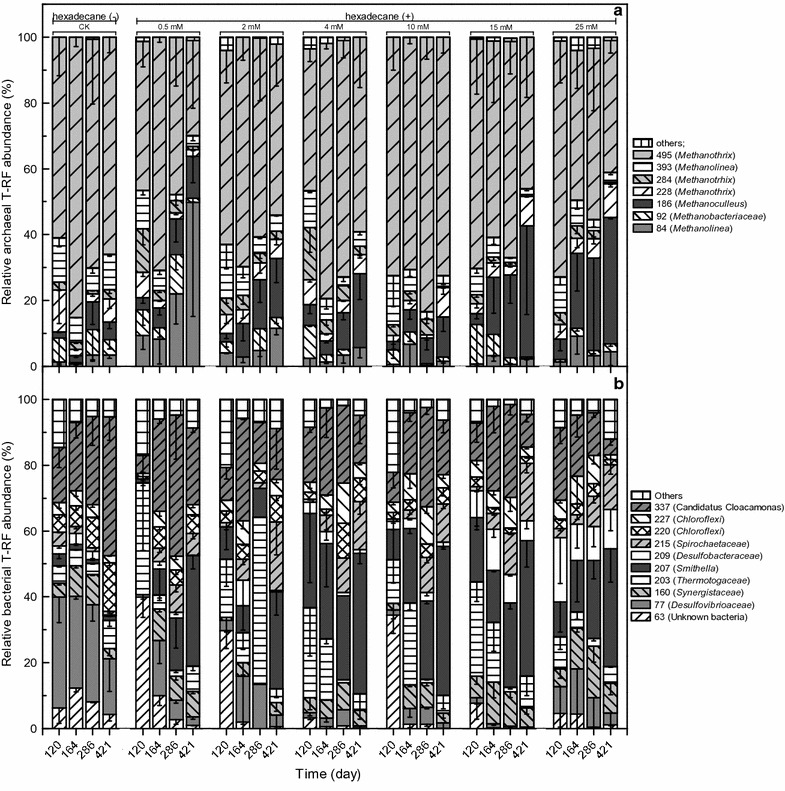
Relative abundance levels of T-RFs of archaeal (**a**) and bacterial (**b**) 16S rRNA genes. CK, culture without hexadecane and sulfate addition; 0.5, 2, 4, 10, 15, and 25 mM represent cultures amended with hexadecane and 0.5, 2, 4, 10, 15, and 25 mM sulfate, respectively. Values represent means and standard errors (*n* = 2–3 replicates). The phylogenetic positions of bacterial phylotypes represented by T-RFs are as referenced in our previous report [34]

The archaeal communities of the cultures with different sulfate concentrations were mainly composed of *Methanothrix* (T-RFs 228, 284 and 495 bp), *Methanolinea* (T-RFs 84 and 393 bp), *Methanobacteriaceae* (T-RF 92 bp), and *Methanoculleus* (T-RF 186 bp) (Fig. 4a; Table 2). *Methanothrix* was one of the dominant phylotypes (Fig. 4a). However, *Methanolinea*, which was represented by the 84-bp T-RF, increased substantially over time and became the predominant phylotype in the 0.5 mM sulfate culture at the end of incubation (Fig. 4a). The *Methanoculleus* populations also increased in all sulfate cultures and became the second dominant phylotype in the 2–25 mM sulfate cultures (Fig. 4a). Pearson correlation analysis revealed that the *Methanoculleus* and *Methanolinea* populations (T-RF 84 bp) were significantly and positively correlated with methane production (*r* = 0.454 and 0.352, respectively, *p* < 0.01) (Table 3). *Methanoculleus* also exhibited a positive correlation with sulfate consumption (*r* = 0.351, *p* < 0.05), whereas *Methanolinea* (T-RF 84 bp) had a significantly negative correlation (*r* = −0.421, *p* < 0.01) (Table 3). ANOSIM indicated that the archaeal communities from the 0.5 and 2 mM sulfate cultures were more similar to each other than cultures in which 4–25 mM sulfate was added (Table 4).

**Table 2:** Archaeal 16S rRNA gene clone libraries

Phylogenetic group	In silico T-RF (No. of clones)^a^			Clone type (GenBank Accession Number)	Closest species	Similarity (%)
	CK	0.5 mM	25 mM			
*Methanosaetaceae*	228 (2)			A4-6 (KJ735834)	*Methanosaeta harundinacea* 6Ac; NR_102896	99
	495 (12)	495 (11)	495 (10)	A18-26 (KJ735844)		96
	791 (1)			A80-6 (KR297252)		82
*Methanomicrobiaceae*		186 (3)	186 (12), 187 (1)	A4-7 (KJ735835)	*Methanoculleus receptaculi* ZC-2; NR_043961	99
			186 (2)	A18-28 (KJ735845)		95
*Methanoregulaceae*		84 (9)		A4-21 (KJ735839)	*Methanolinea tarda* NOBI-1; NR_028163	98
	84 (1), 393 (1)			A80-2 (KJ735846)		97
*Methanobacteriaceae*	93 (1), 95 (6)			A80-8 (KJ735840)	*Methanothermobacter thermautotrophicus* CaT2; AP011952	83
		91 (1), 92 (5)		A4-27 (KJ735841)	*Methanobacterium beijingense* 4-1; AY552778	99

^a^The 16S rRNA gene clone libraries were retrieved from the cultures after 421 days of incubation. CK, the culture without hexadecane and sulfate addition; 0.5 mM: the culture only amended with hexadecane and 0.5 mM sulfate; 25 mM: the culture amended with hexadecane and 25 mM sulfate

**Table 3:** Pearson correlations of T-RFs with methane production and sulfate consumption

Domain	Phylogenetic position	T-RF (bp)	Methane production	Sulfate consumption
Archaeal domain	*Methanolinea*	84	0.454^a^	−0.421^a^
	*Methanolinea*	393	−0.659^a^	−0.089
	*Methanobacteriaceae*	92	−0.073	−0.337^b^
	*Methanoculleus*	186	0.352^a^	0.351^b^
	*Methanosaeta*	228	0.082	0.528^a^
	*Methanosaeta*	284	−0.274^b^	−0.206
	*Methanosaeta*	495	−0.259	0.048
Bacterial domain	Unknown bacteria	63	−0.482^a^	−0.161
	*Desulfovibrionaceae*	77	−0.158	0.047
	*Synergistaceae*	160	−0.068	0.326^b^
	*Thermotogaceae*	203	−0.370^a^	−0.188
	*Smithella*	207	0.532^a^	0.314^b^
	*Desulfobacteraceae*	209	−0.333^a^	0.428^a^
	*Spirochaetaceae*	215	0.715^a^	0.136
	*Chloroflexi*	220	0.467^a^	−0.443^a^
	*Chloroflexi*	227	0.029	−0.061
	*Candidatus* Cloacamonas	337	0.262^b^	−0.499^a^

^a^Correlation is significant at the 0.01 level (2-tailed)

^b^Correlation is significant at the 0.05 level (2-tailed)

**Table 4:** ANOSIM pairwise comparison of similarities among archaeal communities following the addition of different sulfate concentrations

Sulfate concentration (mM)	0.5	2	4	10	15	25
0.5						
2	−0.023					
4	0.156^a^	−0.050				
10	0.319^a^	0.136	−0.015			
15	0.213^a^	0.020	−0.014	0.036		
25	0.150^a^	−0.030	0.025	0.122	−0.001	

^a^Value is significant at the 0.05 level

#### Bacterial community

**Fig. 5:**
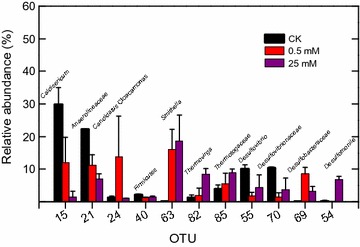
Relative abundance levels of top 11 OTUs. CK, culture without hexadecane and sulfate addition; 0.5 and 25 mM represent cultures amended with hexadecane and 0.5 and 25 mM sulfate, respectively. Values represent means and standard errors (*n* = 2 replicates)

The most common bacterial groups were members of *Smithella* (T-RF 207 bp), *Synergistaceae* (T-RF 160 bp), *Thermotogaceae* (T-RF 203 bp), *Spirochaetaceae* (T-RF 215 bp), *Chloroflexi* (T-RFs 220 and 227 bp), *Candidatus* Cloacamonas (T-RF 337 bp), and unknown bacteria (T-RF 63 bp) (Fig. 4b). These bacterial groups, with the exception of the 63-bp T-RF identified here, have been proposed as the core microbiome of the methanogenic hexadecane-degrading culture M82 without sulfate addition [12]. Members of the core microbiome were positively associated with methane production, except for *Thermotogaceae*, which was negatively associated with methane production (*p* < 0.01) (Table 3). Only *Smithella* exhibited positive associations with methane production and sulfate consumption (Table 3). *Desulfobacteraceae* was negatively associated with methane production but was positively correlated with sulfate consumption (*p* < 0.01) (Table 3). ANOSIM indicated that the bacterial communities could be divided into three clusters corresponding to the 0.5, 2–10, and 15–25 mM sulfate cultures, respectively (Table 5). The bacterial community structure was further evaluated through high-throughput sequencing of the 16S rRNA gene. In total, 318,839 reads with an average length of 413 bp were retrieved from the control and the 0.5 and 25 mM sulfate cultures at the end of incubation (Additional file 2: Table S1). The top 11 operational taxonomic units (OTUs) accounting for 62–75% of the sequences are shown in Fig. 5. Members of *Caldisericum* (an average of 30%), *Anaerolineaceae* (22%), and *Desulfovibrionaceae* (20%, two OTUs) were predominant in the control without sulfate and hexadecane addition (Fig. 5). The dominant phylotypes in the 0.5 mM sulfate addition cultures belonged to *Smithella* (16.1%)*, Candidatus* Cloacamonas (13.8%), *Caldisericum* (12.0%), unclassified *Anaerolineaceae* (11.2%), *Desulfobacteraceae* (8.6%), and *Thermotogaceae* (5.5%), similar to the bacterial T-RFLP profile (Figs. 4b, 5). In contrast, only *Smithella* (18.7%), *Thermotogacea* (8.9%), and *Anaerolineaceae* (7.0%) were retained as the dominant members in the 25 mM sulfate culture (Fig. 5). The relative abundance of *Thermovirga* increased to above 7% in the high-sulfate culture (Fig. 5). Among the four known SRPs (*Desulfomonile*, unclassified *Desulfobacteraceae* and two OTUs in family *Desulfovibrionaceae*), members of *Desulfovibrionaceae* were dominant (19.3–21.5%, two OTUs) in the control (Fig. 5). Unclassified *Desulfobacteraceae* was predominant (7.2–10.0%) in the 0.5 mM sulfate culture, whereas *Desulfomonile* (6.1–7.5%) was predominant in the 25 mM sulfate culture (Fig. 5).

**Table 5:** ANOSIM pairwise comparison of similarities among bacterial communities following the addition of different sulfate concentrations

Sulfate concentration (mM)	0.5	2	4	10	15	25
0.5						
2	0.483^a^					
4	0.996^a^	0.056				
10	0.647^a^	0.013	−0.092			
15	0.995^a^	0.194^a^	0.045	0.090		
25	0.976^a^	0.247^a^	0.287^b^	0.217^b^	0.085	

^a^Value is significant at the 0.05 level

^b^Value is significant at the 0.01 level

## Discussion

### Impacts of sulfate on hexadecane degradation and methane production

The results of this study join those of a previous body of reports demonstrating that methanogenic cultures can degrade alkanes anaerobically in the presence of sulfate [11, 15, 31]. Methane production was enhanced under low-sulfate concentrations (<2–5 mM) [15, 31], whereas significant inhibition was observed at sulfate concentrations greater than 5 mM [31]. Gieg et al. [11] reported that the extent of methanogenic degradation of crude oil alkanes was not affected by sulfate in the 10 mM range, but the methane production rate slightly decreased. The results of this study demonstrated that the rate and extent of methane production were gradually inhibited by increasing sulfate concentration (Fig. 1). However, the presence of excessive sulfate at 25 mM, which is higher than the 21 mM needed for complete conversion of hexadecane to CO_2_ through sulfate reduction, did not completely inhibit methane production (Eq. 2). The concurrent methane production and sulfate reduction in the excessive presence of sulfate may be attributed to the adequate supply of noncompetitive substrates or an abundance of competitive substrates for the syntrophs and methanogens [43]. However, the possibility of cooperation between the incomplete-oxidizing sulfate reducers and methanogens cannot be ignored if incomplete oxidation of organic intermediates into H_2_ or acetate through sulfate reduction presented in the cultures [44]. This study also demonstrated that sulfate reduction increased hydrocarbon degradation efficiency, although the enhancement may not be directly used to address questions of methanogenic and sulfidogenic competition [45, 46]. The increased activity of sulfate reduction after the treatment with increased sulfate concentration probably enhanced H_2_ conversion rate and caused a much lower hydrogen concentration below the threshold value for hydrogenotrophic methanogens [44, 47, 48], which may facilitate the syntrophic alkane oxidation process from a thermodynamics standpoint [18].

The concept of “electron flow” was introduced by Isa et al. [49] to quantify the competition between methanogenesis and sulfidogenesis. The most common explanation for the electron distribution between sulfate reduction and methane production during organic substrate degradation is the effect of the COD/SO_4_^2−^ ratio [49–51], which also includes other factors, such as H_2_S, substrate concentration, and inoculum volume [49]. As expected, electron flux toward sulfidogenesis increased with increasing sulfate concentrations in this study (Fig. 2a). However, unlike most previous work showing how electron flow can be affected by the COD/SO_4_^2−^ ratio [50–53], the relative distribution of electrons between the SRPs and the methanogenic populations depended on the initial sulfate addition and remained constant during methane production (Fig. 2b), which has not been reported before. The results suggested that the concentration of sulfate could be an effective indicator of hexadecane metabolism pathway under mixed electron acceptors conditions. Giving the complex composition of crude oil and diverse electron acceptors present in oil reservoirs, it is also necessary in the future to evaluate the in situ effects of sulfate on the carbon and electron flow during methanogenic crude oil degradation, which could aid in predicting the biomethane potential of crude oil in field trials.

### Impact of sulfate on the methanogenic pathway

The *α*_app_ is a generally accepted index to coarsely estimate the dominant methane production pathway [42], with values above 1.065 and below 1.025 proposed to represent hydrogenotrophic and aceticlastic methanogenesis, respectively, according to recent reports [54, 55]. However, the *α*_app_ value apparently changes according to species and growth conditions, and the values measured in various cultures of hydrogenotrophic methanogens range between 1.031 and 1.077 [56, 57]. Two methanogen genera, *Methanosarcina* and *Methanothrix*, are capable of performing aceticlastic reactions [58], whereas only *Methanothrix* species were observed in this study (Fig. 4a), and the fractionation factor of this genus during aceticlastic methanogenesis is less than 1.01 [56, 59]. Moreover, the acetate concentration was always lower than the threshold value (100 μM) during incubation, indicating that carbon isotope fractionation did not occur during the conversion of acetate to CH_4_ by aceticlastic methanogens [60]. Therefore, the increase in *α*_app_ observed during the incubation of all sulfate addition cultures likely revealed that CO_2_ reduction was the predominant methanogenic pathway, although acetate disproportionation would also contribute to hexadecane degradation. Meanwhile, *α*_app_ decreased gradually with increasing sulfate concentration (Fig. 3c), indicating that sulfate reduction often outcompetes hydrogenotrophic methanogenesis for H_2_ utilization from thermodynamic and kinetic points of view [48, 49].

### Microbial community response to sulfate stress

The aceticlastic *Methanothrix* was the dominant archaea, not only in the hexadecane- and sulfate-containing cultures but also in the control culture in which hexadecane and sulfate were not added (Fig. 4a). The dominant *Methanothrix* did not show a positive relationship with either methane production or sulfate reduction (Table 3). These observations indicated that *Methanothrix* may play a minor role in methanogenic hexadecane degradation, which was not only supported by the aforementioned changes in methanogenic communities but also by increasing *α*_app_ values over time in all sulfate treatments. Regardless, *Methanothrix* may still play a role by leaking H_2_ from acetate degradation to SRPs for sulfate reduction [61]. Both *Methanoculleus* and *Methanolinea* species, which use H_2_/CO_2_ for methanogenesis [62, 63], were positively associated with methane production (Fig. 4a; Table 3). The *Methanoculleus* populations steadily increased with increasing initial sulfate concentrations and became the second most abundant after *Methanothrix* in the 2–25 mM sulfate cultures. This increase could be explained by the fact that *Methanoculleus* may have a greater affinity than other hydrogenotrophic methanogens for H_2_ [64] or the fact that *Methanoculleus* is involved in the syntrophic alkane oxidation process, together with SRPs [34].

*Smithella* is the predominant bacterial phylotype responsible for alkane degradation under methanogenic conditions [33, 34]. Detection of the *ass*A gene and methyl pentadecyl succinic acid in the methanogenic hexadecane-degrading culture has demonstrated that *Smithella* can initiate alkane degradation via fumarate addition [39]. However, no *dsr*-like gene was detected in the binned genome of strain M82_1 [39]. The observation of a positive correlation between *Smithella* abundance and sulfate addition (Fig. 4b; Table 3) may reflect cooperation rather than competition between syntrophic alkane degraders and SRPs, as supported by reported increased hexadecane degradation efficiency with increasing sulfate consumption (Additional file 1: Figure S1). The core bacterial phylotypes affiliated with *Spirochaetaceae*, *Chloroflexi*, *Candidatus* Cloacamonas, and *Thermotogaceae* were proposed to play roles as either secondary degraders during the degradation of hexadecane [12] and terephthalate [65] or contributors to scavenging anabolic products (protein and lipids), presumably derived from detrital microbial biomass [65]. Most of these bacteria were positively correlated with methane production, but their relationships with sulfate reduction were either negative or irrelevant (Table 3), which may reflect the competition of these phylotypes with SRPs.

SRPs in the methanogenic cultures have the capacity to cooperate with methanogens and syntrophic bacteria under sulfate-free conditions but also possess robustness to compete with these organisms through sulfate reduction when sulfate becomes available [66]. The abundance levels of unclassified *Desulfobacteraceae* (OTU 69) and *Desulfomonile* (OTU 54) increased, and these genera became the predominant SRPs in the low- and high-sulfate addition cultures, respectively (Fig. 5). Most members of *Desulfobacteraceae* can utilize sulfate and acetate as electron acceptors and carbon sources, respectively, and some can oxidize organic substrates completely to CO_2_, whereas others perform an incomplete oxidation of organic substrates to acetate [67]. *Desulfomonile* spp., known as dehalogenators, is also capable of H_2_ scavenging, and H_2_ uptake can increase by more than fourfold when sulfate is used as an electron acceptor [47]. These findings suggest that the response of the sulfate reduction bacterial community to sulfate varies according to sulfate concentrations, although their ecophysiological roles in methanogenic hexadecane-degrading cultures in the presence of sulfate require further examination.

## Conclusions

This study not only demonstrated the competition and coexistence of sulfate-reducing and methanogenic populations during anaerobic hexadecane degradation processes, but also revealed a linear positive relationship between the ratio of sulfate reduction/methane production and initial sulfate concentration. The removal efficiency of hexadecane could be enhanced by increased sulfate addition, although the amount of methane accumulation decreased. CO_2_ reduction was the dominant methanogenic pathway, but the activity was inhibited by increasing sulfate addition. Both *Smithella* and *Methanoculleus*, the key alkane degraders and methane producers, could be adapted to the sulfate stress, and different SRP populations were stimulated at various sulfate concentrations. Taken together, our results would help to understand the interactions between methanogenic populations and SRPs during alkane degradation under mixed electron acceptors conditions.

## Methods

### Culture and growth conditions

A methanogenic hexadecane-degrading enrichment culture was derived from oily sludge-contaminated sediment from the Shengli oil field in eastern China [34]. The culture was inoculated (20%, vol/vol) into a modified freshwater medium and dispensed into 600-ml vials under an atmosphere of N_2_ (99.999%) gas, where 100 μl of hexadecane (99%, approximately 0.34 mmol), 2 ml of 2,2,4,4,6,8,8-heptamethylnonane (HMN), and various concentrations of sodium sulfate (giving final concentrations of 0.5, 2, 4, 10, 15 and 25 mM) were added. Neither hexadecane nor sulfate was added to the control culture. The cultures were reduced with Na_2_S·9H_2_O (0.03%), and the pH was adjusted to 7.0 ± 0.2 by the addition of HCl or NaOH solutions prior to incubation. The vials in each set were prepared in triplicate and statically incubated at 35 °C in the dark.

### Chemical methods

Methane was measured using a gas chromatograph (Agilent 7820A, USA) with a thermal conductivity detector, as previously described [12]. The maximum specific methane production rates (*μ*_max_) were calculated [68] from the fitting methane production curves based on a statistical model of Slogistic 1 using ORIGINPRO 8.5 software (OriginLab, USA) The carbon isotopic compositions of CH_4_ and CO_2_ were determined using an IsoPrime100 mass spectrometer (United Kingdom) [34]. The liquid cultures (2–8 ml) were carefully aspirated into tubes using anoxic, sterilized syringes, and were centrifuged at 12,000×*g* for 5 min at 4 °C. The supernatants were collected and stored at −40 °C for future analysis of sulfate and volatile fatty acid (VFA) concentrations. The cell pellets were harvested and stored at −80 °C for microbial community composition analysis (see below). The sulfate concentration was measured using ion chromatography (Metrohm ICS 3000, Switzerland) with a conductivity detector. The liquid samples were diluted 10-fold with deionized water and pretreated using Cleanert IC-RP filtration columns (Agela Technologies, China) before analysis. The eluent contained 30 mM NaOH, and the flow rate was 1.3 ml/min. VFAs were analyzed by gas chromatography (GC) (Agilent 7890A, USA) with a flame ionization detector (FID) and a DB-FFAP column [69]. The residual hexadecane after 421 days of incubation was extracted using a mixture of *n*-hexane and acetone (1:1, v/v) according to a previous report [40]. A final concentration of 300 mg/l pristine was also added before extraction to evaluate the recovery efficiency of the method. The extract was analyzed by GC (Agilent 7890A, USA) using a DB-5MS capillary GC column (30 m × 0.53 mm × 1.5 μm). The GC injector and FID temperatures were set to 280 and 250 °C, respectively. The oven temperature program began at 60 °C for 2 min and increased to 300 °C at a rate of 12 °C per min, where it remained for 10 min. The carrier gas was hydrogen (99.999%) at a flow rate of 36 ml/min. The hexadecane concentration was determined from the external calibration curve generated from *n*-heptadecane, which was calibrated using the linear equation derived from the peak areas.

### Electron flow distribution between sulfate reduction and methane production

The stoichiometric energy-balance equation for the complete oxidation of hexadecane via methanogenesis (Eq. 2) or coupled to sulfidogenesis (Eq. 3) can be written according to previous reports, when cell growth was not taken into consideration [15, 70].

2 $$ {\text{C}}_{ 1 6} {\text{H}}_{ 3 4} + 7. 5 {\text{ H}}_{ 2} {\text{O}} \to 1 2. 2 5 {\text{ CH}}_{ 4} + 3. 7 5 {\text{ CO}}_{ 2} $$

3 $$ {\text{C}}_{ 1 6} {\text{H}}_{ 3 4} + 1 2. 2 5 {\text{ SO}}_{4}^{ 2- } + 2 4. 5 {\text{H}}^{ + } \to 1 2. 2 5 {\text{ H}}_{ 2} {\text{S}} + 1 6 {\text{ CO}}_{ 2} + 1 7 {\text{ H}}_{ 2} {\text{O}} $$

The distribution of electron flow between sulfidogenic and methanogenic reactions can be calculated from the amounts of sulfate reduced (*S*) and methane produced (*M*) [49]. Percent electron flow via methane production was calculated as (*M*) = [*M*/(*M* + *S*)] × 100; percent electron flow via sulfate reduction was calculated as (*S*) = [*S*/(*M* + *S*)] × 100, and the ratio of sulfate reduction and methane production was calculated as S/M.

### Microbial analyses

#### Genomic DNA extraction and purification

Genomic DNA was extracted using a bead-beating method [12]. DNA products were purified with the Promega Wizard DNA Clean-up System (Promega, USA) and were stored at −80 °C.

#### T-RFLP analysis and clone library construction

For T-RFLP analysis, the archaeal and bacterial 16S rRNA gene fragments were amplified using the primer sets A109F/A934R and B27F/B907R, respectively, where the 5′ ends of primers B27f and A934r were labeled with 6-carboxyfluorescein (FAM) [12]. The PCR products were purified with the TIAN Universal DNA Purification Kit (Tiangen, China) and were digested at 37 °C using *Hae*III for bacterial DNA (TakaRa, Japan) and at 65 °C using *Taq*I for archaeal DNA (TakaRa, Japan) [12, 34]. Semi-quantitative DNA fragment analysis was performed as previously reported [12]. The primers and PCR used to construct the archaeal 16S rRNA gene clone library were the same as for the T-RFLP analysis, except that label-free primers were used [12].

### MiSeq and bioinformatics analysis of bacterial 16S rRNA gene fragments

The bacterial primers 515F (5′-GTGYCAGCMGCCGCGGTA-3′) and 909R (5′-CCCCGYCAATTCMTTTRAGT-3′) were used for high-throughput sequencing of 16S rRNA gene PCR amplicons. The PCR mixture and program were described previously [71], and sequencing was performed on the Illumina MiSeq platform. Paired-end reads were merged using FLASh v1.2.9 [72] and were quality-filtered using Trimmomatic v0.32 [73]. Chimeras were removed using the Uchime algorithm and Usearch v 8.1.1861 [74]. The sequence reads in each sample were normalized using daisychopper.pl (http://www.festinalente.me/bioinf/downloads/daisychopper.pl). OTUs were clustered at 97% identity using Qiime v1.9.1 [75]. The phylogenetic affiliations of the representative OTU sequences were assigned using RDP Classifier at a confidence level of 80% [76]. Alpha diversity indices (Chao1, Shannon and Simpson) were calculated using Qiime [75].

### Statistical analysis

Principal coordinate analysis (PCoA) of the community structures was conducted in PAST (http://folk.uio.no/ohammer/past/). The significant differences in community structures between data sets were assessed for significance with ANOSIM using PAST. Correlations between community structures and environmental factors were conducted using the R package Vegan (http://cran.r-project.org/web/packages/vegan/index.html). One-way-analysis of variance (ANOVA), and Pearson’s correlations between T-RFs and environmental factors were conducted using SPSS 17.0 software (IBM, USA). The Pearson correlation coefficient (*r*) and the coefficient of determination (*R* ^2^) were used to evaluate the relationship between the sulfate reduction/methane production ratio and the initial sulfate concentrations, which were also analyzed using SPSS 17.0 software (IBM, USA).

## Additional files

**Additional file 1: Figure S1.** Residual hexadecane contents after 421 days of incubation.

**Additional file 2: Table S1.** High-throughput sequencing of 16S rRNA gene fragments.

## Abbreviations

SRP: sulfate-reducing prokaryote
HMN: 2,2,4,4,6,8,8-heptamethylnonane
VFA: volatile fatty acid
T-RFLP: terminal restriction fragment length polymorphism
FAM: 6-carboxyfluorescein
OTU: operational taxonomic units
PCoA: principal coordinate analysis
ANOVA: one-way-analysis of variance
*μ*_max_: maximum specific methane production rate, month^−1^
δ^13^CH_4_: δ^13^C value of methane
δ^13^CO_2_: δ^13^C value of carbon dioxide
*α*_app_: apparent fractionation factors
