# Supplementary figures and images for: Coexistence and competition of sulfate-reducing and methanogenic populations in an anaerobic hexadecane-degrading culture
Source: Biotechnol Biofuels. 2017 Sep 5;10:207. doi: 10.1186/s13068-017-0895-9 (PMC5584521; doi:10.1186/s13068-017-0895-9)

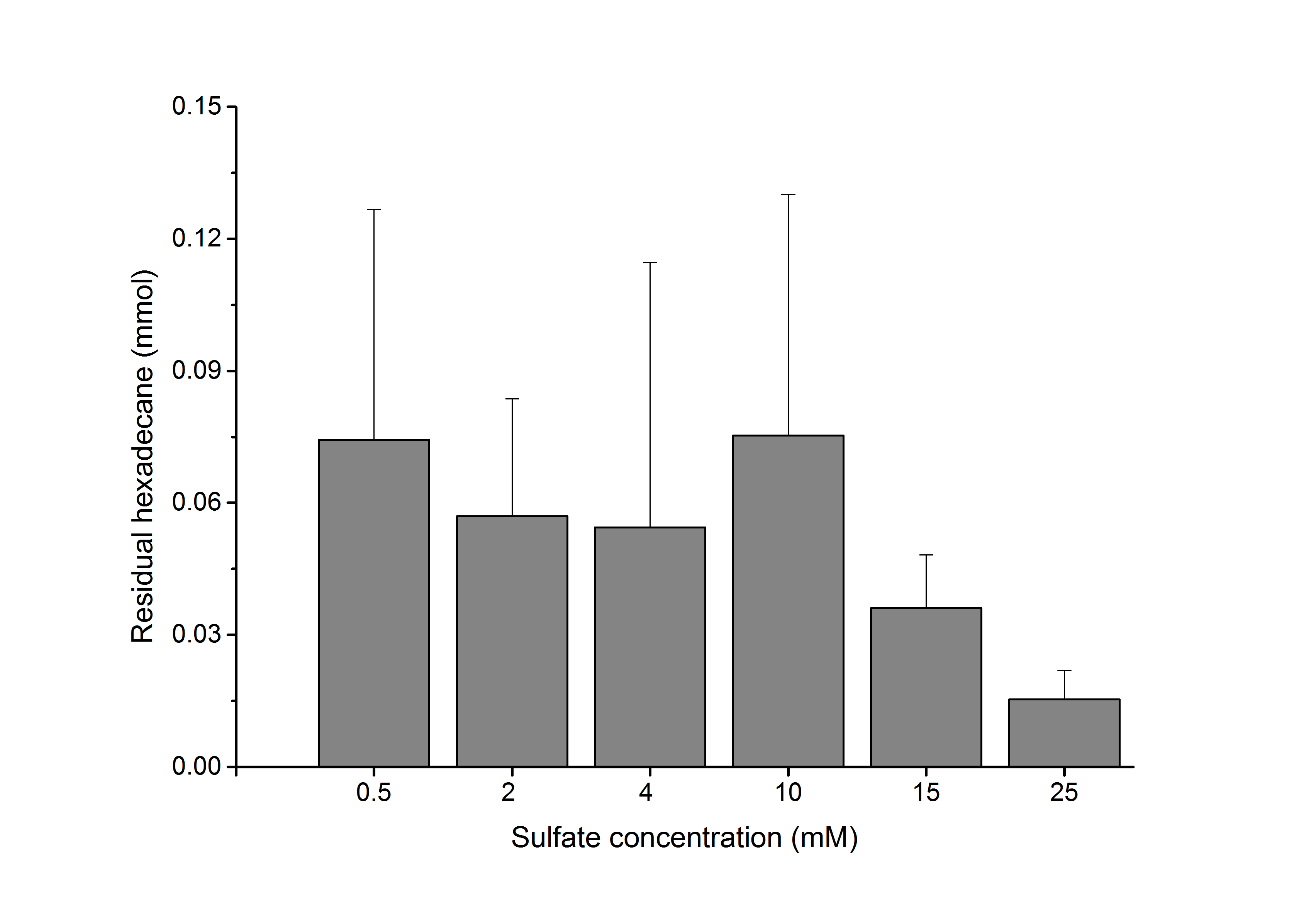


Additional file 1: Figure S1 Residual hexadecane content after 421 days of incubation

Supplement: Supplementary file 1 — Additional file 1: Figure S1. Residual hexadecane contents after 421 days of incubation. [file 13068_2017_895_MOESM1_ESM.docx]
