# Supplementary material for: Coexistence and competition of sulfate-reducing and methanogenic populations in an anaerobic hexadecane-degrading culture
Source: Biotechnol Biofuels. 2017 Sep 5;10:207. doi: 10.1186/s13068-017-0895-9 (PMC5584521; doi:10.1186/s13068-017-0895-9)
Supplement: Supplementary file 2 — Additional file 2: Table S1. High-throughput sequencing of 16S rRNA gene fragments. [file 13068_2017_895_MOESM2_ESM.docx]

Additional file 2: Table S1 High-throughput sequencing of 16S rRNA gene fragments *

| Groups | Sample ID | No. of sequences | Average length (nt) | Observed OTUs* | Simpson* | Shannon* | Evenness* | Chao1* |
| --- | --- | --- | --- | --- | --- | --- | --- | --- |
| CK-1 | K89 | 46992 | 413.2 | 471 | 0.94 | 3.65 | 0.08 | 626.4 |
| CK-2 | K90 | 56321 | 413.2 | 461 | 0.95 | 3.88 | 0.10 | 606.2 |
| 0.5-1 | K91 | 48683 | 414.2 | 464 | 0.97 | 4.17 | 0.14 | 627.8 |
| 0.5-2 | K92 | 67548 | 413.4 | 448 | 0.96 | 4.07 | 0.13 | 630.0 |
| 25-1 | K93 | 57780 | 411.9 | 416 | 0.96 | 3.94 | 0.12 | 590.0 |
| 25-2 | K94 | 41515 | 412.7 | 465 | 0.98 | 4.34 | 0.16 | 652.0 |

^*^: The 16S rRNA gene sequences were retrieved from the cultures after 421 days of incubation. CK-1 and CK-2: the cultures without hexadecane and sulfate addition; 0.5-1 and 0.5-2: the cultures only amended with hexadecane and 0.5 mM sulfate; 25-1 and 25-2: the cultures amended with hexadecane and 25 mM sulfate. Duplicates in each group.

*: these indexes were generated using 19,659 random sequences from each sample and indicate the average values.
